# Replication of tobamovirus RNA

**Published:** 2004-05-01

**Authors:** Masayuki Ishikawa, Yoshimi Okada

**Affiliations:** *) Graduate School of Agriculture, Hokkaido University, Kita 9, Nishi 9, Kita-ku, Sapporo, Hokkaido 060-8589, Japan and CREST, JST, 4-1-8, Honcho, Kawaguchi, Saitama 322-0012, Japan; ***) Professor emeritus, The University of Tokyo

**Keywords:** Tobacco mosaic virus, replication, viral vector

## Abstract

Tobamovirus is a positive-strand RNA virus of plants. Its single-stranded RNA genome replicates *via* the negative-strand RNA. In this review, we describe our current knowledge about viral and host factors associated with tobamovirus RNA replication and discuss the replication mechanisms. We also mention the usefulness of tobamovirus genomes as vectors.

## Introduction

**Fig. 1:**
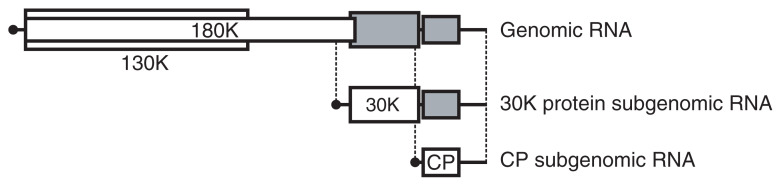
Schematic representation (not to scale) of the ToMV genomic and subgenomic RNAs. The open and gray boxes represent the translatable and non-translatable open reading frames, respectively.

The genus *Tobamovirus* includes many virus species, such as *Tobacco mosaic virus* (TMV), *Tomato mosaic virus* (ToMV), *Cucumber green mottle mosaic virus* (CGMMV), *Sunnhemp mosaic virus* (formerly TMV-Cc), *Odontoglossum ringspot virus* (ORSV), *Youcai mosaic virus* (recently proposed to be the same species as the crucifer-infecting tobamovirus TMV-Cg), *Tobacco mild green mosaic virus* (TMGMV), and the rakkyo-infecting tobamovirus (TMV-R).1) The tobamovirus genome consists of an approximately 6,400-nucleotide, messenger-sense RNA with a 7-methylguanosine cap at the 5’-terminus. The tobamovirus genome encodes proteins of 130 kDa (the 130 K protein), 180 kDa (the 180 K protein: a readthrough derivative of the 130 K protein), 30 kDa (the 30 K protein) and the coat protein (CP), reading from the 5’- to the 3’-end.2),3) Genomic RNA replication proceeds *via* full-length negative-strand RNA, which is complementary to the genomic RNA. The 30 K protein and CP are synthesized by translation of the respective subgenomic RNAs, which are 3’-co-terminal with the genomic RNA (Fig. 1). The subgenomic RNAs are synthesized by internal initiation from the full-length negative-strand RNA, in a process called transcription.

In 1986, *in vitro* transcription systems that allowed the production of infectious tobamovirus RNA from cloned full-length cDNA copies were established for TMV by Dawson’s group4) and for ToMV by Okada’s group.5) With these experimental systems, reverse genetics could be used to determine the function of tobamovirus-encoded proteins and RNA sequence elements. As a result, molecular studies on the mechanisms of tobamovirus RNA replication have progressed dramatically.

## Tobamovirus-encoded proteins involved in viral RNA replication

Initially, both the 130 K and 180 K proteins were thought to be involved in the replication of tobamovirus RNA, based on the observation that they were the only proteins that were translated directly from the genomic RNA prior to replication. The most convincing experimental evidence that both these proteins are involved in tobamovirus replication was obtained using artificially constructed mutants. Ishikawa *et al*. constructed various ToMV mutants that expressed either the 130 K protein or the 180 K protein by introducing mutations at or near the amber termination codon for the 130 K protein gene. The mutants with the intact gene for the 130 K protein, but with the defective gene for the 180 K protein were not infectious. On the other hand, the mutant, in which the amber termination codon of the 130 K protein gene was replaced with a tyrosine codon, which resulted in the production of only the 180 K protein, replicated in tobacco leaves or protoplasts, albeit at a low level of multiplication.6),7) This result suggests that the 180 K protein can carry out all the functions of the 130 K protein. Nonetheless, the balanced expression of the 130 K and 180 K proteins (at a ratio of about 10–20:1) is necessary for the efficient replication of ToMV RNA. On the other hand, the mutant in which the genes for the 30 K protein and CP were deleted replicated normally in tobacco protoplasts.8) This result clearly demonstrates that the 30 K protein and CP are dispensable for replication. Indeed, the 30 K protein and CP have been shown to be required for cell-to-cell and long-distance movement of the virus, respectively.9)–11)

Osman and Buck purified a crude membrane-bound RNA-dependent RNA polymerase (RdRp) from ToMV-infected tomato leaves by sucrose density gradient centrifugation. This polymerase, which contained the viral 130 K, 180 K proteins, as well as several host proteins and double-stranded ToMV RNA, incorporated labeled ribonucleotides into both double-stranded and single-stranded ToMV RNA. The membrane-bound polymerase became dependent on exogenously added ToMV RNA when treated with micrococcal nuclease. This template-specific ToMV RdRp was inhibited by the addition of antibodies against the 130 K or 180 K proteins. These results indicate that the 130 K and 180 K proteins are components of the tobamovirus RdRp.12)

**Fig. 2:**
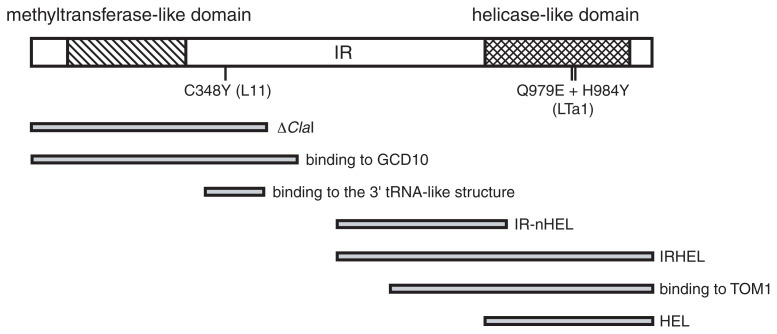
Arrangement of the methyltransferase-like and helicase-like domains of the tobamovirus 130 K protein. The uppermost thick box represents the 130 K protein (1115 amino acids). The methyltransferase-like and helicase-like domains are shaded with diagonal lines and crosshatching, respectively. The intervening region (IR) is located between these two domains. The positions of the causal mutations in ToMV mutants L_11_ and Lta1 are indicated below the 130 k protein. The other subregions of the 130 K protein are shown by thin boxes, and are discussed in detail in the text.

The amino acid sequence of the read-through region of the 180 K protein contains a motif that is found in RdRp.13),14) Thus, the 180 K protein probably provides the catalytic activity for the synthesis of tobamovirus RNA. The 130 K protein contains an N-terminal domain that is involved in the 5’-end capping reaction, and which is related to methyltransferase and guanylyltransferase 15) (Fig. 2). It has been demonstrated that isolated membranes from TMV-infected plants or insect cells that express the 130 K protein, are capable of forming the guanylated 130 K protein and synthesizing m^7^GTP using *S*-adenosylmethionine as the methyl group donor. These reactions are considered to be important for the capping of TMV RNA.16),17) The 130 K protein also has a C-terminal helicase-like domain18),19) (Fig. 2). The helicase activity is probably essential for tobamovirus RNA replication, in unwinding the duplexed or structured RNA that is formed during RNA synthesis. Polypeptides that contain the TMV helicase domain and that are expressed in *E. coli* are capable of unwinding duplexed RNA,20) which confirms the predicted helicase function of this domain. The amino acid sequences of these three domains (the methyltransferase-like, helicase-like, and polymerase-like domains) are highly conserved among the animal and plant viruses that belong to the alpha-like virus superfamily.21)–24)

In addition to the enzymatic activities discussed above, the 130 K and 180 K proteins also have the ability to bind specifically to the 3’-noncoding region of the ToMV RNA *in vitro*. A region immediately downstream of the core methyltransferase domain of the 130 K protein is responsible for this binding (Fig. 2). A ToMV mutant with a mutation in this region that abolishes the specific binding of the 130 K protein to the 3’-noncoding region of ToMV RNA, was unable to multiply in tomato protoplasts. Therefore, it seems likely that this binding is essential for ToMV RNA replication.25)

Some of the replication-defective 130 K- and 180 K-defective mutants can be trans-complemented by self-replicatable helper tobamoviruses.26)–28) The ability of defective mutants to be trans-complemented depends on the removal of the read-through portion of the 180 K protein-coding sequence, which suggests that this region contains an element that interferes with trans-complementation.28) Lewandowski and Dawson found that the RNA region corresponding to the N-terminal one-third of the 130 K protein (the ‘**Δ***Cla*I’ region in Fig. 2) needed to be translated for efficient trans-complementation.29) These characteristics assist in the appropriate selection of replication template. The *cis* action of replication proteins may represent a general strategy for template selection in monopartite positivestrand RNA viruses.30),31) Knapp *et al*. found that replication-defective TMV mutants that expressed the intact 130 K protein, in which the read-through region of the 180 K protein gene was deleted, could be transcomplemented by a replication-competent mutant that expressed the functional 180 K protein but not the 130 K protein; these transcomplemented TMV mutants accumulated at moderate levels both in protoplasts and in plants. However, these defective viruses could not be transcomplemented by the wild-type TMV. Knapp *et al*. also found that replication-defective TMV mutants that expressed only the Δ*Cla*I region of the 130 K protein, with the remainder of the replication protein-coding region being deleted, could be transcomplemented by the wild-type TMV. These mutants accumulated at high levels in protoplasts but not in plants, which suggests that these defective viruses cannot move in plants. These results may explain why defective tobamoviruses are rarely found in nature.32)

Watanabe *et al*. prepared template-specific TMV RdRp by solubilization of the P30 fraction of TMV-infected tobacco leaf extracts with the anionic detergent taurodeoxycholate, followed by immunoaffinity purification using antibodies against the TMV replication proteins. This RdRp preparation contained a heterodimer of the 130 K and 180 K proteins.33) Yeast two-hybrid analysis demonstrated that the helicase domain polypeptide (the HEL polypeptide in Fig. 2) interacts with the IR-nHEL polypeptide that encodes the C-terminal half of the intervening region and the N-terminal portion of the helicase domain (Fig. 2).34) Mutations that disrupt this interaction render the virus non-infectious.34) Furthermore, it has been shown that the HEL and IRHEL polypeptides (Fig. 2) oligomerize, to form ringlike complexes that display six-sided symmetry.20) The replication complexes of many alpha-like viruses are small spherical bodies that bud into the organelle lumen. These spherical bodies are 50–100 nm in diameter, surrounded by membranes, and contain replication templates and multiple copies of the viral replication proteins. 35) Although a replication complex in the form of a spherical body has not been reported for tobamoviruses, the tendency of TMV replication proteins to oligomerize suggests that the tobamovirus replication complex also contains multiple replication proteins.

The 130 K and/or 180 K proteins are also known to be involved in the elicitation of the *N* gene-mediated hypersensitive response in tobacco plants.36)

## The RNA sequence elements required for tobamovirus RNA replication and subgenomic RNA transcription

The tobamovirus genomic RNA harbors 5’- and 3’-noncoding regions of approximately 70 and 200 nucleotides, respectively. *A priori*, these terminal regions have been considered to be important for the replication, translation, and stability of tobamovirus RNA,37) and the accumulating data support this idea as discussed below.

The 3’-proximal 100-nucleotide region of tobamovirus RNA is folded into a tRNA-like structure, and is a substrate for cellular tRNA-modifying enzymes, which include aminoacyl-tRNA synthetase and (ATP, CTP):tRNA terminal nucleotidyltransferase. Indeed, tobamovirus RNA is aminoacylated with histidine or valine *in vitro*.37) Osman *et al*. have demonstrated that the tRNA-like structure is important for *in vitro* minus-strand RNA synthesis by the template-dependent ToMV RdRp.38) Furthermore, deletions of the 3’-terminal sequences or the addition of extra non-viral sequences results in the loss or reduction of *in vivo* infectivity of tobamovirus RNA,4),5) which indicates the importance of intact 3’-terminal structures. However, a recent study with *Turnip yellow mosaic virus*, which belongs to the *Tymovirus* genus in the alpha-like virus superfamily, suggests that aminoacylation of the genomic RNA itself is not an absolute requirement for RNA replication.39)

In the 3’-noncoding region of the tobamovirus RNA, three consecutive pseudoknot structures, each of which is composed of two double-helical segments, are present immediately upstream (i.e., in the 5’ direction) of the tRNA-like structure.40) To elucidate the biological functions of the pseudoknots, Takamatsu *et al*. introduced several deletion mutations in this region. The mutant in which five out of the six double-helical segments were deleted, such that the pseudoknot structures were no longer formed, was still able to replicate. This indicates that the complete set of pseudoknot structures is not essential for replication. However, it has been shown that either deletion of the double-helical segment immediately upstream of the tRNA-like structure or a base substitution that destabilizes the helix results in loss of infectivity.41) Gallie *et al*. have demonstrated that this pseudoknot region enhances the translation of the genomic RNA,42) which raises the possibility that the loss of infectivity is caused not only by defects in RNA replication, but also by reduced translation of the TMV-coded proteins. Although the host-encoded eukaryotic elongation factor 1A43) and p102 (HSP101)44),45) have been shown to bind to the pseudoknot region, the importance of the interaction between the pseudoknot region and these proteins in the translation and replication of tobamovirus RNA have not been determined.

Ishikawa *et al*. constructed ToMV-derived chimeras, in which the 3’-noncoding region was replaced by the corresponding regions of either TMV RNA, CGMMV RNA, or TMV-Cc RNA. ToMV, TMV, and CGMMV RNA have tRNA-like structures that are histidylated, and TMV-Cc RNA has a tRNA-like structure that is valylated. The three chimeric viruses were able to multiply in both tobacco protoplasts and plants, although the multiplication levels of the latter two constructs were lower than that of ToMV.46) In order to define the limit of this flexibility, the 3’-noncoding region of the genomic RNA of *Brome mosaic virus* (BMV), which belongs to the *Bromovirus* genus in the alpha-like virus superfamily, and that has a tRNA-like structure that is tyrosylated, was used to construct ToMV chimeras. Although the level of virus accumulation was very low, the chimeric virus was able to replicate in protoplasts.47) This result suggests that either the recognition of the 3’-noncoding structure by RdRp is not so stringent or that the RdRp recognizes a common structural feature that is shared by these viral 3’-terminal sequences. This flexibility may be permitted, since the template for replication is also strictly selected in *cis* by the 130 K protein, as discussed above.

The 5’-noncoding region of ToMV RNA lacks internal guanosine residues and contains CAA repeats. It is believed that the complementary sequence of the 5’-noncoding region carries information that is important for the initiation of genomic RNA synthesis. Takamatsu *et al*. analyzed the infectivity of ToMV mutants that had nucleotide deletions in the 5’-noncoding region. Mutants with deletions of about ten nucleotides, whereby these ten nucleotides were located in the region between nucleotides 10 and 71 (referred to as “short deletion mutants”), maintained infectivity in tobacco protoplasts, while the deletion of nucleotides 2 to 8 caused a loss of infectivity.48) Watanabe *et al*. inoculated one of the short deletion mutants into Samsun tobacco plants, which represent a systemic host for ToMV. Although some of the inoculated plants showed severe mosaic symptoms, the remaining plants showed no symptoms. The severity of symptoms paralleled the levels of virus accumulation. Analysis of the progeny virus from the plants with severe mosaic symptoms revealed that they were revertants that had re-acquired CAA repeats. Thus, this region probably controls the ability of ToMV to systemically infect whole plants.49) Wells *et al*. have shown that the 5’-noncoding sequence of TMV contains a translational enhancer, and that the specific binding of HSP101 protein to this region is necessary for this enhancement.45) The involvement of HSP101 in tobamovirus replication and spread in plants has not been determined.

Subgenomic RNA transcription is initiated internally from the full-length negative-strand RNA. Sequences upstream and downstream of the transcription initiation sites (−35 to +10 for the 30 K protein subgenomic RNA, and –69 to +12 for the CP subgenomic RNA) are necessary for transcription.50) In addition to these *cis*-acting sequences, genomic position is known to have a significant impact on the transcription of subgenomic RNAs.51)

## Host proteins involved in tobamovirus RNA replication

The multiplication of eukaryotic positive-strand RNA viruses involves a complex interplay between the virus genomes, virus-coded proteins, host proteins, and intracellular membranes.52) The identification of host factors that are involved in virus multiplication is therefore important in understanding the molecular mechanisms of virus multiplication. Although our current knowledge about these host factors is insufficient, several host proteins have been implicated in tobamovirus multiplication using different approaches.

Osman and Buck purified taurodeoxycholate-solubilized ToMV RdRp from infected tomato tissues, and found that the 56-kDa, 54-kDa, and 50-kDa host proteins were associated with the RdRp. The 56-kDa host protein cross-reacts with the anti-yeast GCD10 protein antibody, and this antibody inhibits RdRp activity.53) The yeast GCD10 protein has been shown to interact with the methyltransferase-like domain polypeptide in the yeast two-hybrid system.54) In yeast, the GCD10 protein is required for 1-methyladenosine modification and maturation of initiator methionyl-tRNA, although it had been considered to be a subunit of the eukaryotic translation initiation factor (eIF)-3.55) The function of this protein in ToMV RNA replication remains to be revealed. The TMV RdRp that was prepared by Watanabe *et al*. contained host proteins of 220-kDa and 34-kDa,33) the identities of which have not been revealed.

Ishikawa *et al*.56) and Ohshima *et al*.57) isolated mutants of *Arabidopsis thaliana*, in which TMV-Cg multiplication was reduced to low levels. These mutants carry the independent recessive mutations, *tom1* (*tobamovirus multiplication 1*) and *tom2*. Since these mutations are recessive and active at the protoplast level, their wild-type gene products (TOM1 and TOM2) probably function to support the multiplication of TMV-Cg within host cells.57),58) Furthermore, since these mutations affect the multiplication of ToMV but do not affect the multiplication of either *Turnip yellow mosaic virus* or *Turnip crinkle virus* (genus *Carmovirus*),56),57) it seems likely that TOM1 and TOM2 function through specific interactions with tobamovirus-encoded factors.

Positional cloning studies have demonstrated that the *TOM1* gene encodes a protein with seven predicted transmembrane regions.59) On the other hand, analyses of the *tom2* mutant identified two distinct genes, *TOM2A* and *TOM2B*, which are involved in intracellular tobamovirus multiplication. TOM2A is predicted to be a four-pass transmembrane protein, and TOM2B appears to be a small basic protein.60) Using yeast two-hybrid assays (the Sos recruitment system), TOM1 was found to interact with the helicase domain polypeptides of TMV replication proteins (Fig. 2).59) This result suggests that TOM1 tethers the 130 K and 180 K proteins to the membranes. Furthermore, the split ubiquitin assay demonstrated that TOM2A interacts not only with TOM1, but also with itself.60) This observation implies that TOM1 and TOM2A form multimers on membranes. This type of multimerization, together with the multimerization of the 130 K and 180 K proteins themselves *via* the helicase domain,20) may help to form a replication complex that contains multiple viral replication protein molecules, as is observed commonly for alpha-like viruses.35)

Although each of the three *tom1* mutants that have been independently isolated to date carries a mutation that is predicted to result in a complete loss of function, TMV-Cg and ToMV can still multiply at low levels in these mutant plants. Yamanaka *et al*. further mutagenized one of the *tom1* mutants, to obtain a mutant line in which the multiplication of TMV-Cg and ToMV was completely inhibited. Genetic analysis revealed that this mutant carried another recessive mutation (named *tom3*) in addition to *tom1*. The loss of the *TOM3* gene alone did not noticeably affect TMV-Cg or ToMV multiplication. Positional cloning of the *TOM3* gene has revealed that it encodes a protein that is closely related to TOM1. Therefore, TOM1 and TOM3 probably have parallel and essential roles in tobamovirus multiplication.61)

Subcellular localization analysis using GFP fusions of TOM1 and TOM2A has shown that these proteins are localized mainly on tonoplasts (vacuolar membranes). Iodixanol density gradient centrifugation analysis demonstrated that TOM2A was almost exclusively present on membranes that fractionated to the low buoyant density fractions that contained the tonoplasts. TOM1 fractionated to the low buoyant density fractions, but also to higher buoyant density fractions that contained multiple types of membranes, including the endoplasmic reticulum (ER) and Golgi body. The fractionation of lysates of ToMV-infected tobacco protoplasts showed that the fractionation patterns of the 130 K and 180 K proteins and viral RdRp activity were similar to that of TOM1. Based on these results, Hagiwara *et al*. proposed that TOM1 tethers the 130 K and 180 K replication proteins to membranes, and TOM2A facilitates the binding of the replication proteins to TOM1 and/or the formation and maintenance of the replication complex.62) Given that TOM2A is barely detectable in the higher buoyant density fractions, it may not be absolutely essential for tobamovirus replication complex formation. Alternatively, functional homologues of TOM2A may be present in the higher buoyant density fractions.

*Tm-1* is a semi-dominant tomato gene that inhibits the intracellular multiplication of ToMV. *Tm-1* inhibits ToMV multiplication in protoplasts, even in the presence of actinomycin D, which suggests that the molecules required for resistance are not induced but are pre-existing in uninfected cells.63) A ToMV mutant (Lta1), which has the ability to multiply in *Tm-1* tomato plants, carries mutations that result in amino acid changes (Gln 979 to Glu and His 984 to Tyr) in the helicase domain of the replication proteins (Fig. 2).64),65) This result suggests that the 130 K and/or 180 K proteins are the targets of *Tm-1*. It is possible that the *Tm-1* gene product is a dominant-negative allele of a gene that is required for ToMV RNA replication. The *Tm-1* gene has not been identified to date, and the details of the mechanisms of inhibition of ToMV multiplication have yet to be revealed. With respect to map-based cloning of this gene, *Tm-1* has been mapped to a position close to the ribosomal RNA genes on chromosome II, and DNA markers with tight linkage to the *Tm-1* locus have been obtained.66)

Hamamoto *et al*. reported that a single amino acid substitution (Gln 979, one of the mutation sites in Lta1, to Ile) in the 130 K and 180 K proteins of ToMV altered host specificity. This ToMV mutant (TLIle) was unable to replicate in tomato protoplasts, whereas it replicated normally in tobacco protoplasts.67) The 979th amino acid residue may play an essential role in the interaction between the 130 K and 180 K proteins and some host-encoded factor that is required for ToMV RNA replication in tomato cells. The corresponding host factor in tobacco may be more flexible, allowing it to interact successfully with the 130 K and 180 K proteins of the TLIle mutant.

## The role of membranes in tobamovirus replication

After invasion of the host cell and uncoating, the genome of the eukaryotic positive-strand RNA virus is translated, to produce the virus-coded replication proteins. The set of replication proteins recruits the genomic RNAs as replication templates to the cytoplasmic surfaces of intracellular membranes, isolates them from the translation machinery and other cytoplasmic macromolecular machinery, and forms replication complexes. 35) Isolation of the replication template from the translation machinery may be important, because the movement of ribosomes from the 5’-terminus would inhibit the movement of RdRp from the 3’-terminus of the replication template.68) In the replication complexes, negative-strand RNA is synthesized; this is probably followed by the exclusive synthesis of positive-strand genomic and subgenomic RNAs.35) Isolation of the negative-strand RNA in the replication complexes from the cytoplasmic compartment may be important to escape from RNA silencing, as mentioned below. In protoplasts that are infected with ToMV, negative-strand RNA accumulation ceases at an early stage of infection (6 h post inoculation), while positive-strand RNAs continue to accumulate during later periods after infection (16–18 h post inoculation).7) This observation suggests that one of the essential host components of the ToMV replication complex is limited in number.

The membranes upon which the replication complexes are formed vary from virus to virus. However, for each virus, the replication complex is formed on specific organelle membranes, which suggests a specific molecular interaction between the replication proteins and the membranes. For tobamoviruses, the membranes on which the replication complexes reside remain controversial. Immunostaining tobamovirus-infected cells with anti-replication protein antibodies has demonstrated that the replication proteins are localized to cytoplasmic granular inclusion bodies,69) in ER-associated inclusion, designated X-bodies,70) and on the ER.71) However, recent analyses suggest that only a fraction of the ToMV replication proteins are associated with membranes and involved in replication, while the remaining proteins are not associated with membranes, and have no detectable ToMV RNA synthesis activity *in vitro*. The latter fraction is composed of the majority of the replication protein pool during the late stages of infection, when most of the immunocytological analyses have been performed.62) Therefore, the immunostaining signals detected in these studies may represent mainly non-membrane-bound replication proteins. Iodixanol density gradient centrifugation analysis of extracts from ToMV-infected protoplasts has shown that the replication proteins are associated with membranes that are fractionated at low-buoyant-density fractions that contain tonoplasts, and also with membranes that are fractionated at higher-buoyant-density fractions that contain various membranes such as ER and Golgi body. The fractionation pattern of membrane-bound replication proteins resembled that of TOM1. These results suggest that the ToMV replication complex is formed on TOM1-bearing membranes including tonoplasts. 62)

Consistent with the importance of membranes for the formation and maintenance of replication complexes, the solubilization of replication complex-containing membranes results, in most cases, in the loss of RdRp activity, or in RdRp that can synthesize only the complementary strand RNA to exogenously added positive-or negative-strand RNA templates of the homologous virus. In exceptional cases, detergent-solubilized RdRp catalizes the complete replication cycle of exogenously added homologous viral genomic RNA.72),73) As discussed above, Osman and Buck obtained template-containing ToMV RdRp, which produced a large amount of ss ToMV RNA and a trace amount of ds ToMV RNA, by solubilization of the membrane-bound ToMV RdRp with taurodeoxycholate. 53) This result suggests that taurodeoxycholate solubilizes the ToMV replication complex, while maintaining partial functionality.

Recently, Komoda *et al*. established a cell-free translation-replication system for tobamovirus RNA. This system utilizes lysates of non-infected plant protoplasts, from which the vacuoles have been removed by stepwise density gradient centrifugation. Importantly, this lysate contains membranes, since the ultracentrifugation and gel filtration steps that remove membranes are omitted in the preparation of the lysate. In this lysate, ToMV RNA is translated to produce the 130 K and 180 K proteins, followed by the addition of ^32^P-labelled ribonucleoside triphosphates. This reaction produces internally ^32^P-labeled ss ToMV genomic and subgenomic RNAs and the ds replicative-form RNA, in a similar pattern to that observed for ToMV-infected protoplasts.74) Therefore, this system appears to reproduce the *in vivo* processes of: (1) selection of genomic RNA as the replication template by the 130 K and 180 K proteins; and (2) formation of the replication complexes on membranes. This system will facilitate studies on the mechanisms of tobamovirus replication.

## Tobamovirus replication protein as a suppressor of post-transcriptional gene silencing, and as an enhancer of viral cell-to-cell movement

Plant cells have the ability to degrade RNA in response to specific ds or highly-structured RNAs that have nucleotide sequence homology to the target RNA. This phenomenon, called RNA silencing, has also been found in various eukaryotic organisms, including animals and fungi, and is thought to function to protect the cells against invasion by viruses or transposable elements. During RNA silencing, dsRNA is first degraded by enzymes that are related to *Drosophila* DICER, to generate 21–25-nucleotide RNA molecules, which are called ‘small interfering (si) RNAs’. This siRNA is incorporated into an enzyme complex, which is named the ‘RNA-induced silencing complex’ (RISC), and the RISC degrades target RNA in a sequence-specific manner. siRNA is also thought to act as a primer of target dsRNA synthesis by the host RdRp, which results in amplification of the RISC.75)

In order to multiply successfully, viruses have evolved a variety of strategies to escape RNA silencing.75) In the natural replication cycles of positive-strand RNA viruses, the negative-strand RNA is stringently sequestered from the cytoplasmic space, where the RNA silencing machinery resides, and is strictly isolated within membrane-bound replication complexes. This appears to be necessary to avoid triggering RNA silencing. 35) In addition, many viruses encode proteins that inhibit RNA silencing.75) In the case of ToMV, the 130 K replication protein suppresses RNA silencing. Kubota *et al*. established tobacco plants that were transformed with a cauliflower mosaic virus 35*S* RNA promoter-driven green fluorescent protein (GFP) gene cassette, in which GFP expression was suppressed by RNA silencing. The transgenic tobacco plants were susceptible to ToMV, and showed mosaic symptoms, as did non-transformed tobacco plants. In the leaves of the ToMVinfected transgenic tobacco plants that showed mosaic symptoms, RNA silencing was suppressed (i.e., GFP fluorescence was observed) within the light-green area where ToMV accumulated, while RNA silencing was still active in the dark-green area, in which there was no ToMV accumulation. In contrast, the attenuated ToMV strain L_11_, which has a causal mutation in the coding region of the 130 K protein (Fig. 2), showed reduced ability to suppress RNA silencing. Importantly, these authors demonstrated that the 130 K protein that was expressed by the Agro-infiltration method acted as a suppressor of RNA silencing. Further analysis indicated that the 130 K protein did not prevent the accumulation of siRNA, but did inhibit the formation of RISC. This result suggests that the 130 K protein inhibits the utilization of siRNA for RISC formation.76)

After replication of the genomic RNA within an initially infected cell, the genomic RNA moves to the neighboring cells *via* the plasmodesmata, in a process that requires the 30 K protein. Hirashima *et al*. have shown that a chimeric TMV carrying the helicase domain region of TMV-R can replicate and express the 30 K protein in protoplasts, although this virus cannot move from cell to cell.77) Further analyses with revertants and chimeric viruses suggest that incompatibility between the helicase domain of TMV-R replication proteins and the intervening region (‘IR’ in Fig. 2) of the TMV replication proteins produces this phenotype.78) Mutations in the BMV 2a polymerase gene that generate a similar phenotype have also been reported.79) Tamai and Meshi demonstrated that the ToMV 30 K protein-facilitated cell-to-cell movement of GFP was more active when ToMV RNA replication occurred in the cell that expressed the 30 K protein and GFP than in the absence of ToMV RNA replication.80) These observations suggest that tobamovirus RNA replication influences cell-to-cell movement of macromolecules, which is dependent upon the 30 K protein. The mechanism behind this linkage of replication and movement is yet to be revealed.

## Utilization of tobamoviruses as vectors for foreign gene expression

As a consequence of the extremely high efficiencies of multiplication and gene expression of tobamoviruses, the tobamovirus genome has been utilized as a vector for foreign gene expression in plants.81) The first tobamovirus-based vector was constructed by Takamatsu *et al*., and consisted of a simple gene replacement vector, in which the coat protein gene was replaced by the bacterial chloramphenicol acetyltransferase (CAT) gene. This chimeric virus replicated, spread from cell to cell, and produced active CAT within the inoculated tobacco leaves. However, due to the lack of the CP, the virus could not spread systemically. 82)

In order to create a vector that was capable of systemic infection, Dawson *et al*. constructed a chimeric TMV, in which the coat protein-coding region was replaced with a foreign gene, and a second CP mRNA promoter, followed by a CP gene, was inserted behind the foreign gene sequence. However, upon infection of plants, homologous recombination deleted the region between the twice-repeated subgenomic RNA promoter sequences, which included the foreign gene.83) To reduce the loss of foreign genes due to homologous recombination, improved vectors have been constructed by substituting the sequence downstream of the foreign gene with the ORSV (for the vector TB2),84),85) TMGMV U5 (for the vector 30B),86) or ToMV (for the vector TTO1)87) sequences that encompass the CP subgenomic RNA promoter, CP gene, and 3’-noncoding region. These vectors are sufficiently stable for the production of foreign proteins in plants. To further improve the 30B vector, Toth *et al*. employed a DNA shuffling method, to specifically mutagenize the 30 K protein gene, and obtained mutant vectors with improved movement properties.88) Hori and Watanabe also constructed a ToMV-based vector that is similar to 30B (referred to as TocJ). This vector has proven to be useful for foreign protein expression in some *Solanaceous* plant species.89)

One attractive approach to the production of vaccines is to display immunogenic epitopes on the surfaces of plant virus particles. Tobamoviruses are suitable for this purpose, since large quantities of pure virus particles can easily be purified from infected plants. Turpen *et al*.90) and Koo *et al*.91) have inserted the malarial epitope and the murine hepatitis virus spike glycoprotein epitope at an internal site and in a C-terminal region of the CP, respectively; both sites are predicted to be exposed to the surface of the virion. These viruses replicate, spread systemically in plants, and successfully display immunogenic epitopes on the surfaces of assembled tobamovirus particles.90),91) However, the insertion of polypeptides into these regions of the CP does not always permit systemic infection and virion assembly. 92),93) To overcome these limitations, Hamamoto *et al*. have designed a vector in which a polypeptide-coding sequence is fused to the C-terminus of the CP *via* a leaky termination codon. The resulting virus multiplies systemically in host plants, and the wild-type CP and fusion protein co-assemble into virus particles.94) Using this vector, the angiotensin I-converting enzyme inhibitor peptide,94) epitopes from the influenza virus hemagglutinin and human immunodeficiency virus type I envelope protein,95) and the malarial epitope,90) have been displayed successfully on the surfaces of recombinant tobamovirus particles.

In addition to the production of foreign proteins or peptides, pioneering work has been performed with tobamovirus vectors with respect to demonstrating virus-induced gene silencing96) and to engineering plant metabolic pathways for the production of novel compounds *via* epigenetic expression of foreign genes.97) Recently, cDNA libraries of *A. thaliana* were constructed in both the sense and antisense orientations using a tobamovirus vector. These libraries will be useful in identifying the functions of a large number of *A. thaliana* genes.98)
